# Dataset for Bluetooth 5.1 Direction of Arrival with non Uniform Rectangular Arrays

**DOI:** 10.1016/j.dib.2021.107576

**Published:** 2021-11-14

**Authors:** Nicolò Ivan Piazzese, Michele Perrone, Danilo Pietro Pau

**Affiliations:** a STMicroelectronics, Catania, Italy; b STMicroelectronics, Agrate Brianza, Italy

**Keywords:** Direction of arrival, Bluetooth 5.1, Rectangular antenna arrays, URA8, MUSIC, Pseudo-spectrum

## Abstract

This paper presents a dataset for Bluetooth 5.1 direction of arrival (DoA). The dataset was generated with a specifically designed mathematical model of a non-uniform rectangular antenna array. The Python source files that generated the dataset are also provided. The dataset was conceived as a starting point for developing and validating DoA algorithms for real-life scenarios. Unlike other datasets, it contains Bluetooth signals with not only varying intensity of additive white Gaussian noise, but also coherent interfering signals with random DoA coordinates. The dataset is divided into two branches, one consisting of pure sinusoidal tones and the second comprised of baseband Bluetooth signals. Since the codebase which generates the data is included, this dataset has a high reuse potential, and it can be modified to suit also other types of signals or different array topologies.

**Specifications Table**

**Table:** 

Subject	Computer Networks and Communications
Specific subject area	Telecommunications and Bluetooth signals
Type of data	Dataset files: *.csv files
	Source code for dataset generation: *.py files
How data were acquired	Mathematical simulation of complex signals. Python source files that generated the data are included
Data format	Raw
Parameters for data collection	Useful signal azimuth and elevation (degrees), useful signal to noise ratio (SNR) (dB), useful signal to interfering signals ratio (SIR) (dB), frequency offset between useful signal and interfering signals (interf. offset) (MHz), residual Bluetooth receiver offset (res. offset) (KHz)
Description of data collection	Data was generated with a mathematical model of the signal and of the rectangular antenna array. The simulated signal is a baseband Bluetooth 5.1 signal with constant tone extension. The simulated receiving array is a rectangular array with 8 patch antennas. Many different combinations of data parameter values were considered (SNR, SIR, interf. offset, res. offset)
Data source location	Data was generated at ST Microelectronics, Catania, Italy
Data accessibility	Repository name: Mendeley Data
	Direct URLs to data:
	• Part 1: https://doi.org/10.17632/zjn2zxcgyz.1
	• Part 2: https://doi.org/10.17632/gysjfpvxpm.1
	• Part 3: https://doi.org/10.17632/nnv3vpy4y3.1
	• Part 4: https://doi.org/10.17632/362szwsnc4.1
	**Please note**: you need to collect all four parts in one folder. Then, you can then use an extraction software of your choice, e.g. 7-Zip (https://www.7-zip.org/), to recreate the entire directory structure of the dataset.

## Value of the Data

**Fig. 1:**
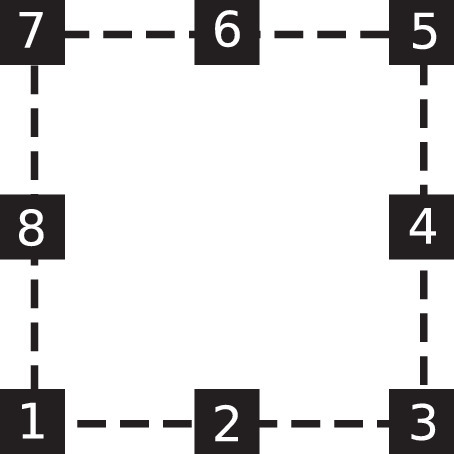
Geometrical configuration of the non-uniform rectangular array with 8 patch elements.

This dataset simulates the interaction of Bluetooth 5.1 signals with the URA8 array topology. The latest Bluetooth 5.1 standard has introduced important features to aid direction-finding solutions [1]. URA8 is an antenna array composed of eight elements equally spaced on a square edge. This type of array is thoroughly analyzed in [2] and is shown in Fig. 1. The mathematical model of the array is presented in detail in Section 3.1.

• The dataset described in this paper is useful because it provides a valuable tool for the development and validation of DoA algorithms for Bluetooth 5.1 signals. It contains coherent interference with respect to the useful signal, which is always present in real-life scenarios. Since DoA algorithms are usually tested and compared on sinusoidal signals, both sinusoidal signals and Bluetooth signals are included in this dataset. Moreover, the dataset provides the signals in the form of IQ samples, which is how they are outputted by modern Bluetooth 5.1 devices.
• Anyone who develops Bluetooth 5.1 tracking applications can benefit greatly from this dataset, because the simulated data are comparable to those obtained through actual measurements. The data have been compared with real-world IQ samples generated by an STMicroelectronics transceiver prototype, fully compliant with the Bluetooth 5.1 standard. This prototype has been equipped with an eight-sensors patch antenna array, with the same topology as the mathematical model of the array shown in Fig. 1 and described in Section 3.1. Experimental results have shown that the measured IQ samples match the signals modeled in the presented dataset with a signal to noise ratio (SNR) of 60dB and a with signal to interference ratio (SIR) below 60dB, showing a mean error equal to 0 and a standard error deviation around 1%. The non-zero value of the standard error deviation is due to a residual stochastic uncompensated frequency offset on the measured IQ samples.
• By including the Python codebase that generates the dataset alongside the dataset itself, the data can be modified and expanded at wish. For example, more than two interfering signals could be added, the topology of the array could be modified, or the number of simulated measurements could be increased. The latter aspect is especially valuable when considering the training of artificial neural networks, which require large quantities of data to generalize appropriately.

## 1 Data Description

The dataset consists of .csv data, along with .py Python source files that are used to generate the data. It is divided into two branches, each one corresponding to the type of signal which interacts with the rectangular array. The first branch is devoted to radio frequency (RF) sinusoidal signals, while the second one contains Bluetooth 5.1 signals. Each branch is self-contained within its own folder. The content of the PureTone_data/ and BLE_5_1_data/ folders is described in Sections 2.1 and 2.2 respectively. The Python code that was used to generated the dataset, contained in folders PureTone_code/ and BLE_5_1_code/, is described in Section 3.3.



### 1.1 RF 2.4GHz pure tone branch

The directory structure is shown below. The data is organized in a series of simulated tests, each contained within its own folder, whose name always begins with Test_*/. Each test folder then contains $N_{\phi }\cdot N_{\theta }$ sub-folders, where $N_{\phi }$ and $N_{\theta }$ are the number of possible azimuth and elevation angles of the useful signal, respectively. The description of every dataset parameter is reported in Table 1, and is valid for both dataset branches. The range of each PureTone dataset branch parameter is reported in Table 2.

**Table 1:** Description of the dataset parameters, valid for both dataset branches.

Parameter	Abbreviation	Description	Measure unit
<$a_{i}$>	Iof	Frequency offset between the useful signal and interfering signals	MHz
<$b_{i}$>, <$c_{i}$>	SIR	Useful signal to interfering signals power ratio	dB
<$d_{i}$>	SNR	Useful signal to white Gaussian noise power ratio	dB
<$\hat{e_{i}}$>	rfo	Average residual frequency offset at the Bluetooth receiver	kHz
<$e_{i}$>	roff	Residual frequency offset at the Bluetooth receiver	hHz
<$f_{i}$>	CTE	Constant tone extension duration	μs
<$g_{i}$>, <$h_{i}$>	cte	Constant tone extension ON/OFF (1/0)	/
<$n_{i}$>	m or meas	Simulated measurement number	/
<$p_{i}$>	DR	Data rate	Mbits/s
<$q_{i}$>	Switching	Switch time between successive antenna elements	μs
<hh>, <mm>	/	Hours and minutes of data generation	/
<DD>, <MM>, <YYYY>	/	Day, month and year of data generation	/
<$\phi_{i}^{0}$>, <$\theta_{i}^{0}$>	sig_azim, elv	Azimuth and elevation angle of the useful signal	Degrees (${}^{\circ }$)
<$\phi_{i}^{1}$>, <$\theta_{i}^{1}$>	iaz, iel	Azimuth and elevation angle of the first interfering signal	Degrees (${}^{\circ }$)
<$\phi_{i}^{2}$>, <$\theta_{i}^{2}$>	iaz, iel	Azimuth and elevation angle of the second interfering signal	Degrees (${}^{\circ }$)

**Table 2:** Dataset parameters value range for the PureTone branch.

Parameter	Value range	Measure unit
<$a_{i}$>	{0, 2}	MHz
<$b_{i}$>, <$c_{i}$>	{6, 12}	dB
<$d_{i}$>	{12, 30, 60}	dB
<$\phi_{i}$>, <$\theta_{i}$>	$\phi_{i}\in \left[0,359\right]$ (1${}^{\circ }$ step), $\theta_{i}\in \left[0,90\right]$ (3${}^{\circ }$ step)	${}^{\circ }$
<$\phi_{i}^{1}$>, <$\theta_{i}^{1}$>	Same as <$\phi_{i}^{0}$>, <$\theta_{i}^{0}$>, random distribution	${}^{\circ }$
<$\phi_{i}^{2}$>, <$\theta_{i}^{2}$>	Same as <$\phi_{i}^{0}$>, <$\theta_{i}^{0}$>, random distribution	${}^{\circ }$



There are three types of .csv files for each sig_azim<$\phi_{i}$>_elv<$\theta_{i}$> folder, all of them using UTF-8 encoding and comma as separator. **The following description is valid for both dataset branches:**

• doares_saz<$\phi_{i}$>_sel<$\theta_{i}$>.csv files contain the DoA of the useful signal, estimated by the Multiple signal classification algorithm (MUSIC), which is described in Section 3.2. They are meant to serve as a ground truth for performance comparison with other DoA algorithms. An example of such file is shown in Table 3. The column headers and their abbreviations correspond to the parameters described in Table 1. The results obtained by MUSIC are contained in res_azim (resulting azimuth) and res_elev (resulting elevation) columns.

**Table 3:** Example of a doares_saz<$\phi_{i}$>_sel<$\theta_{i}$>.csv file.

meas	sig_azim	sig_elev	CTE	iaz	iel	res_azim	res_elev
0	0.000000	0.000000	[0 0]	[305 16]	[16 44]	324.000000	9.000000
1	0.000000	0.000000	[0 0]	[305 16]	[16 44]	321.000000	5.000000
0	0.000000	0.000000	[1 1]	[292 169]	[44 44]	292.000000	6.000000
1	0.000000	0.000000	[1 1]	[292 169]	[44 44]	273.000000	9.000000
0	0.000000	0.000000	[1 1]	[337 355]	[21 33]	329.000000	9.000000
1	0.000000	0.000000	[1 1]	[337 355]	[21 33]	345.000000	9.000000
• info_saz<$\phi_{i}$>_sel<$\theta_{i}$>_<hh>_<mm>_of_<DD>_<MM>_<YYYY>.csv files are useful in the case that one does not want to rely on the hierarchy of the directory tree to read the key dataset parameters. Their generic structure is shown in Table 4. They contain the useful signal angular position range (minimum, maximum, and step), the position of the useful signal, the frequency offset <$a_{i}$>, the signal to noise ratio <$d_{i}$> and the first interfering signal to useful signal ratio <$b_{i}$>. The parameters correspond to those described in Table 1.

**Table 4:** Generic content of info_saz<$\phi_{i}$>_sel<$\theta_{i}$>_<hh>_<mm>_of_<DD>_<MM>_<YYYY>.csv files.

**Test started at<hh> <mm> <DD> <MM> <YYYY>**	
**Useful signal**	
useful signal test ranges	azimuth [0 359 1] elevation [0 90 3]
useful signal position	azimuth <$\phi_{i}^{0}$> elevation <$\theta_{i}^{0}$>

**Interfering signals**	
first interf. signal frequency offset from useful signal	<$a_{i}$> MHz

**SNR and SIR**	
SNR	<$d_{i}$> dB
first interf. signal SIR	<$b_{i}$> dB
• X_m<$n_{i}$>_*.csv files contain the 8×70 $X_{IQ}$ complex-valued IQ matrices, which result from the interaction of the signals and the rectangular antenna array. These files do not contain any header. Each 8×1 column is a complex-valued IQ vector ${x_{IQ}}^{T}$. The complex numbers are in the format shown in Table 5

**Table 5:** Example of a complex-valued IQ vector ${x_{IQ}}^{T}$ contained in X_m<$n_{i}$>_*.csv files.

1.859307540261690983e+00-1.614217293859837865e-01j
1.483198132423238835e+00-3.605448754221074470e-01j
9.662254468967348409e-01-4.818835170878809637e-01j
1.644941300650671900e+00-1.455036290828832235e-01j
8.238801969038891393e-01+5.024682120440079336e-01j
9.591105763646003979e-01-6.146509479381243035e-02j
9.636106402553353822e-01-2.972847799272813618e-01j
9.085768220898262637e-01+5.888629266394146411e-01j

### 1.2 Baseband Bluetooth 5.1 signal branch

The directory structure is very similar to the PureTone branch and is reported below. The difference consists in the greater number of parameters due to the presence of the residual frequency offset and the constant tone extension (CTE), which are typical of Bluetooth 5.1 signals. Table 1 provides a description for each parameter, while Table 6 reports the value range that can be assumed by each parameter. The three types of .csv files within each sig_azim<$\phi_{i}$>_elv<$\theta_{i}$> folder are identical with respect to the PureTone dataset branch.

**Table 6:** Dataset parameters value range for the BLE_5_1 branch.

Parameter	Value range	Measure unit
<$a_{i}$>	{0}	MHz
<$b_{i}$>, <$c_{i}$>	{3, 6, 12, 18, 24, 30, 36, 48, 54, 60}	dB
<$d_{i}$>	{30, 48, 60}	dB
<$\hat{e_{i}}$>	{-3, 0, +3}	kHz
<$e_{i}$>	$e_{i}\in \left[-3,+3\right]$, Gaussian distribution	hHz
<$f_{i}$>	{1200}	μs
<$g_{i}$>, <$h_{i}$>	{0, 1} (OFF/ON)	/
<$p_{i}$>	{1}	Mbit/s
<$q_{i}$>	{1, 2}	μs
<$\phi_{i}$>, <$\theta_{i}$>	$\phi_{i}\in \left[0,359\right]$ (1${}^{\circ }$ step), $\theta_{i}\in \left[0,90\right]$ (3${}^{\circ }$ step)	${}^{\circ }$
<$\phi_{i}^{1}$>, <$\theta_{i}^{1}$>	Same value range as <$\phi_{i}^{0}$>, <$\theta_{i}^{0}$>, random distribution	${}^{\circ }$
<$\phi_{i}^{2}$>, <$\theta_{i}^{2}$>	Same value range as <$\phi_{i}^{0}$>, <$\theta_{i}^{0}$>, random distribution	${}^{\circ }$



## 2 Experimental Design, Materials and Methods

The dataset is based on a mathematical model of a non-uniform rectangular antenna array with 8 elements. The following sections provide a description of the mathematical model of the array, of the impinging signals, and of their Python implementation which is provided with the dataset.

### 2.1 Mathematical model

https://www.overleaf.com/project/617a8bcbbeb847848f1fd78c The array model corresponds a rectangular array of N=8 numbered antenna elements, evenly spaced at a distance $d=\frac{\lambda }{2.5}$ on a square edge, as shown in Fig. 1. Because of the lack of a central element, this array is non-uniform.

The rectangular array receives M signals $s_{i}\left(n\right)$, incident with angles

(1) $$\left(\theta_{1},\phi_{1}\right),\left(\theta_{2},\phi_{2}\right),...,\left(\theta_{m},\phi_{m}\right),...,\left(\theta_{M},\phi_{M}\right)$$

where $\theta_{m}$ and $\phi_{m}$ are the m-th elevation and azimuth angles of each signal. The signals have also a defined power $P_{1},P_{2},P_{m},...,P_{M}$.

The array response matrix A describes the interaction of the impinging signals with the rectangular antenna array. For the topology of the array shown in Fig. 1, the array response matrix is

(2) $$A=[a{\left(\theta_{1},\phi_{1}\right)}^{T},a{\left(\theta_{2},\phi_{2}\right)}^{T},...,a{\left(\theta_{M},\phi_{M}\right)}^{T})]$$

where the array response vectors a(θ,ϕ) are defined as:

(3) $$a\left(\theta ,\phi \right)=\left[\begin{array}{c} 1\\ e^{-j\gamma (\theta )(dcos(\phi ))}\\ e^{-j\gamma (\theta )(d2cos(\phi ))}\\ e^{-j\gamma (\theta )(2dcos(\phi )+dsin(\phi ))}\\ e^{-j\gamma (\theta )(2dcos(\phi )+2dsin(\phi ))}\\ e^{-j\gamma (\theta )(dcos(\phi )+2dsin(\phi ))}\\ e^{-j\gamma (\theta )(d2sin(\phi ))}\\ e^{-j\gamma (\theta )(dsin(\phi ))} \end{array}\right]$$

with $d=\frac{\lambda }{2.5}$ and

(4) γ(θ)=2πsin(θ)

As mentioned in Section 2, the dataset is subdivided into two branches. For the **Bluetooth 5.1 dataset branch**, the signals $s_{i}\left(n\right)$ are a baseband model of the signals at the analog to digital converter (ADC) of the Bluetooth receiver. Apart from the frequency offset, the carrier translation and the RF impairments are negligible for the study of the DoA. Regarding the frequency offset between the transmitter and the receiver, the majority of the offset is corrected at the automatic frequency corrector (AFC). However, a small part of this offset typically remains, which we indicate as $f_{off}$. This residual offset is able to impact negatively the estimation of the DoA. Our mathematical model of BLE 5.1 signals is that of binary Gaussian frequency shift keying (GFSK) modulated signals:

(5) $$s_{i}\left(n\right)=e^{j\cdot \alpha (n)}\cdot e^{\left(2\cdot \pi \cdot f_{off}\cdot n\right)/F_{s}}$$

(6) $$\alpha \left(n\right)=\frac{2\pi \cdot F_{dev}}{F_{s}}\cdot \sum_{k=0}^{n}\sum_{i}b_{i}p\left(k-iN_{s}\right)$$

(7) $$F_{dev}=\frac{DR\cdot h}{2}$$

where DR is the data rate, $f_{off}$ is a possible frequency offset error, $F_{s}=16MHz$ is the sampling frequency at the receiver’s ADC, n represents the discrete-time index, h is the modulation index, p(n) is the symbol pulse as defined in [3], and $b_{i}\in \left\{1,-1\right\}$ is the binary symbol to be transmitted. As a mathematical model for the baseband CTE, Eq. 5 with all binary symbols $b_{i}$ equal to 1 is used.

The baseband samples at the Bluetooth receiver, running at a proper sample period $T_{s}$ without ADC impairments can be expressed as:

(8) $$X_{T_{s}}=A\cdot \left[\begin{array}{cccc} P_{1} & 0 & ... & 0\\ 0 & P_{2} & ... & 0\\ 0 & ... & ... & 0\\ 0 & 0 & ... & P_{M} \end{array}\right]\cdot \left[\begin{array}{c} s_{1}\\ s_{2}\\ ...\\ s_{M} \end{array}\right]+n$$

where $s_{1},s_{2},...,s_{M}$ are the complex impinging signals, generated by using Eq. 5 with a sampling period $T_{s}$ and $n={\left[n_{1},n_{2},...,n_{8}\right]}^{T}$ is the noise vector at the analog to digital converter (ADC), with zero mean and variance $\sigma^{2}$, which is related to the SNR of the useful tag signal. The noise vector is comprised of AWGN noise, which approximates the noise of the receiver, and does not take into account the interfering signals. When one of the $s_{i}$ signals is a CTE, all binary symbols are set to 1, otherwise they are selected randomly.

Considering the IQ sampling process of the Bluetooth receiver and calling $T_{switch}$ and $T_{sample}=1/F_{s}$ the switch slot duration and the sampling slot duration respectively, both set to 1 μs or 2 μs, the $X_{T_{s}}$ data are down-sampled with a factor of $round(\left(T_{switch}+T_{sample}\right)/T_{s})$, thus obtaining the IQ samples in form of $X_{IQ}$, which is a complex matrix with size $N\times N_{samp}$.

Because of the filtering chain, the power of the adjacent channels, alternate channels and all remaining channels is strongly attenuated in comparison to the reflections of useful signal. This means that, in the BLE 5.1 model, all of the interfering signals are in fact the reflections of the useful signal itself.

In the case of **pure tone dataset branch**, the signals $s_{i}\left(n\right)$ are defined as

(9) $$s_{i}\left(n\right)=e^{j\cdot 2\pi \cdot \left(F_{c}+ch\right)\cdot \left(n/F_{s}\right)}$$

where $F_{c}=2.4GHz$, ch is the channel spacing (which is an integer multiple of 2MHz), and $F_{s}$ is the sampling frequency, set to $F_{s}=F_{c}\cdot 32$. In this model, we do not decimate the $s_{i}$ signals.

The IQ samples are obtained as

(10) $$X_{IQ}=A\cdot \left[\begin{array}{cccc} P_{1} & 0 & ... & 0\\ 0 & P_{2} & ... & 0\\ 0 & ... & ... & 0\\ 0 & 0 & ... & P_{M} \end{array}\right]\cdot \left[\begin{array}{c} s_{1}\\ s_{2}\\ ...\\ s_{M} \end{array}\right]+n$$

where n is the AWGN noise at the antenna elements. Please note that the noise n which is added in the pure signal model is much more wideband when compared to the n noise of the Bluetooth model, because the BLE model is a baseband model, while the pure tone signal is an RF model. Therefore, the signal to noise ratios (SNRs) obtained with the two models are not comparable. This is because the SNR depends on the bandwidth of the noise channel.

### 2.2 The MUSIC algorithm

In addition to the modeled RF 2.4GHz and Bluetooth 5.1 signals, this dataset provides a ground truth for the development and testing of different algorithms. For this purpose, the Multiple signal classification (MUSIC) algorithm [4] was implemented and applied to the simulated dataset signals. MUSIC is a signal subspace method for DoA estimation, which exploits the eigen-structure of the autocorrelation matrix of the signal to find the signal sub-space and noise sub-space. The MUSIC pseudo-spectrum P(ϕ,θ) is obtained in following way:

(11) $$P\left(\phi ,\theta \right)=\frac{1}{a{\left(\phi ,\theta \right)}^{H}\cdot Q_{n}\cdot Q_{n}^{H}\cdot a\left(\phi ,\theta \right)}$$

where a(θ,ϕ) are the array response vectors defined in Eq. 3 and $Q_{n}$ the eigen vectors of the noise sub-space. The DoA estimate of the source signal is then obtained by finding the peak in the above defined pseudo-spectrum.

### 2.3 Python implementation

*Note on licensing.* The Python implementation that is shipped with this dataset is free software: you can redistribute it and/or modify it under the terms of the GNU General Public License as published by the Free Software Foundation, either version 3 of the License, or (at your option) any later version. If you make improvements to the code, you are invited to share those changes with the community.

*File structure and dependencies.* The Python implementation is written in Python 3 and is contained inside the following *.py files:



The following dependencies are needed: os, time, numpy, scipy and matplotlib.

The following paragraphs describe the content of each Python module by making also reference to the mathematical equations of the previous sections.

*The algorithmic module.* The algorithmics_URA8.py module is common to both branches of the dataset. It contains the definition of the conventional steering vector for URA8 as defined in Eq. 3 and the implementation of the MUSIC algorithm as described in Section 3.2. The function which generates the steering vector is the following:



The URA8 steering vector from Eq. 3 is also defined inside the MUSIC implementation:



The MUSIC pseudo-spectrum from Eq. 11 is then estimated:



*The signal model modules.* For the Bluetooth 5.1 branch, the signals are modeled inside the e2e_URA8_BLE.py module. The frequency deviation from Eq. 7 is implemented as



The baseband signals $s_{i}\left(n\right)$ with the frequency offset $f_{off}$, as defined in Eqs. 5 and 6, are implemented as



The directions of the incident signals from Eq. 1 and the array response vectors from Eq. 3 are generated in



**Fig. 2:**
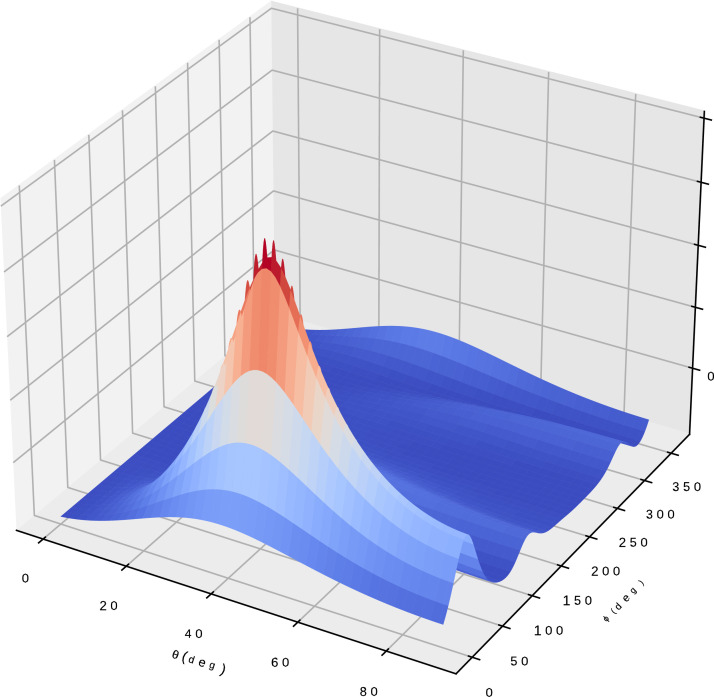
MUSIC pseudo-spectrum plotted by the DoA view modules.

The array response matrix from Eq. 8 is implemented in the following lines:



For the PureTone branch, the signals are modeled inside the e2e_URA8_PureTone.py module. The signals $s_{i}\left(n\right)$ from Eq. 9 are implemented as follows:



The array response vector from Eq. 3, which comprise the array response matrix from Eq. 2 are then computed in the following lines:



The $X_{IQ}$ sample matrix from Eq. 10 is then obtained:



*The dataset generator modules.* The dataset is generated by the e2e_URA8_BLE_DSgen.py and the e2e_URA8_PureTone_DSgen.py modules. Both modules do not accept console arguments. The Bluetooth 5.1 dataset generator allows for the setting of the following parameters before being launched:



A similar approach is used for the parameters of the PureTone dataset generator:



*The testing modules.* The e2e_URA8_BLE_DOAview.py and e2e_URA8_PureTone_DOAview.py modules are meant as an aid for the setting of the dataset parameters prior to its actual generation. Their output is a 3D plot of the MUSIC pseudo-spectrum. The following code is taken from the e2e_URA8_BLE_DOAview.py module. It gives the possibility of manually setting the following parameters:



The e2e_URA8_PureTone_DOAview.py gives the possibility of manually setting the following parameters:



An example of the plots created by the modules is shown in Fig. 2.

## Ethics Statement

The authors declare that the data presented in this article did not involve any use of human subjects, animal experiments nor data collected from social media platforms.

## CRediT Author Statement

**Nicolò Ivan Piazzese:** Conceptualization, Methodology, Software, Formal analysis, Investigation, Writing – original draft, Writing – review & editing; **Michele Perrone:** Conceptualization, Validation, Data curation, Writing – original draft, Writing – review & editing; **Danilo Pietro Pau:** Conceptualization, Validation, Writing – original draft, Writing – review & editing, Supervision, Project administration.

## Declaration of Competing Interest

The authors declare that they have no known competing financial interests or personal relationships which have, or could be perceived to have, influenced the work reported in this article.

## References

[1] Pau G., Arena F., Gebremariam Y.E., You I. (2021). Bluetooth 5.1: An analysis of direction finding capability for high-precision location services. Sensors.

[2] Jin L., li L., Wang H. (2008). 2008 International Conference on Microwave and Millimeter Wave Technology.

[3] Rappaport T. (2002).

[4] Stoica P., Nehorai A. (1988). ICASSP-88., International Conference on Acoustics, Speech, and Signal Processing, volume 4.

